# Mismatch Negativity Latency and Cognitive Function in Schizophrenia

**DOI:** 10.1371/journal.pone.0084536

**Published:** 2014-04-16

**Authors:** Christian Kärgel, Gudrun Sartory, Daniela Kariofillis, Jens Wiltfang, Bernhard W. Müller

**Affiliations:** 1 Clinic for Psychiatry and Psychotherapy, University of Duisburg-Essen, Essen, Germany; 2 Department of Psychology, University of Wuppertal, Wuppertal, Germany; 3 Institute of Forensic Psychiatry, University of Duisburg-Essen, Essen, Germany; 4 Clinic for Psychiatry and Psychotherapy, University of Göttingen, Göttingen, Germany; UNLV, United States of America

## Abstract

**Background:**

The Mismatch Negativity (MMN) is an event-related potential (ERP) sensitive to early auditory deviance detection and has been shown to be reduced in schizophrenia patients. Moreover, MMN amplitude reduction to duration deviant tones was found to be related to functional outcomes particularly, to neuropsychological (working memory and verbal domains) and psychosocial measures. While MMN amplitude is thought to be correlated with deficits of early sensory processing, the functional significance of MMN latency remains unclear so far. The present study focused on the investigation of MMN in relation to neuropsychological function in schizophrenia.

**Method:**

Forty schizophrenia patients and 16 healthy controls underwent a passive oddball paradigm (2400 binaural tones; 88% standards [1 kHz, 80 db, 80 ms], 11% frequency deviants [1.2 kHz], 11% duration deviants [40 ms]) and a neuropsychological test-battery. Patients were assessed with regard to clinical symptoms.

**Results:**

Compared to healthy controls schizophrenia patients showed diminished MMN amplitude and shorter MMN latency to both deviants as well as an impaired neuropsychological test performance. Severity of positive symptoms was related to decreased MMN amplitude to duration deviants. Furthermore, enhanced verbal memory performance was associated with prolonged MMN latency to frequency deviants in patients.

**Conclusion:**

The present study corroborates previous results of a diminished MMN amplitude and its association with positive symptoms in schizophrenia patients. Both, the findings of a shorter latency to duration and frequency deviants and the relationship of the latter with verbal memory in patients, emphasize the relevance of the temporal aspect of early auditory discrimination processing in schizophrenia.

## Introduction

The attenuation of the mismatch negativity (MMN) has been established as a highly reliable biological finding in schizophrenia over the last decades [1], [2]. The MMN, first described by Näätänen et al. [3], is an event related potential (ERP) elicited by the detection of rare, slightly deviant, tone events within a sequence of highly frequent standard tones. A necessary requirement for the MMN generation is thought to be the development of a sensory memory trace representing the regular sensory input immediately before the occurrence of the deviant event [4]. Rare tone events may deviate with regard to various stimulus features from the standard events, for example to frequency, duration, intensity or location [5]. The MMN has been found to be elicited mainly independently from attentional resources (for examples of attentional modulation of the MMN see Müller et al. [6] and Rissling et al. [7]) and is typically obtained while participants are concurrently engaged in a non-demanding task. The “sensory memory-trace” interpretation of the MMN has been challenged by suggestions of an adaptation processes that may contribute to its generation through fresh-afferent neuronal activity [8]. Another recent theory on the MMN incorporates ideas of the generation of stimulus predictions and their violation [9] and suggests that these prediction signals may be generated independently at different levels of cortical processing [10].

The MMN amplitude reduction in schizophrenia varies with type of stimulus deviance [11]. While MMN reduction to frequency deviant tones may develop over the course of the illness [11]–[13], those to duration- and intensity-deviant tones have been shown from early on [13]. The question as to the heritability of the dysfunction is as yet unresolved. Some studies reported similar deficits in unaffected family members [14], [15] but a study in non-symptomatic co-twins of patients did not corroborate this finding [16].

With regard to symptom domains in schizophrenia, relations to MMN amplitude deficits have not been found consistently. Whereas a number of authors reported negative symptoms to be associated with MMN deficits [17]–[20], results of recent studies point at an association with positive symptoms in particular, hallucinations [21]–[23]. In either case, the more distinct MMN deficits, the more pronounced the positive or negative symptoms. However, Umbricht and Krljes' [2] meta-analysis reported no relationship between MMN amplitudes and clinical symptom domains in case of most of the included studies.

Cognitive deficits are considered a core feature of schizophrenia and have been confirmed by numerous studies [24], [25]. Recently, increased attention has been given to the relationship between MMN measures and impairments of cognitive function. Baldeweg et al. [4] reported significant correlations between MMN amplitude reduction to duration deviants and cognitive function in the domains of verbal fluency, executive function and episodic memory. These findings have been replicated for verbal fluency [26] and executive function [27]. Associations between MMN amplitude to duration deviants and measures of visual attention [28] and social cognition [29] have been reported recently. Kawakubo et al. [30] found amplitude to duration deviants with phoneme deviants (but not to sinusoidal tones) related to verbal memory performance in schizophrenia patients. Additionally, Kiang et al. [31] found an association between MMN deficits to duration deviants and verbal memory. In a recent study Miyanishi et al. [32] reported current source density measures to duration MMN in the frontal cortex to be related to poor working memory performance in schizophrenia patients. Conversely, Lin et al. [33] found no significant relationship between MMN parameters and neuropsychological measures in schizophrenia. Apparently, the association between deficits in MMN parameters and neuropsychological performance is not limited to schizophrenia patients. A recent study reported attenuated MMN amplitude to duration deviants to be related to diminished semantic fluency and greater self-rated functional disability in late-life depressives [34]. In patients with mild cognitive impairment reduced MMN amplitude to duration deviants was found to be associated with poor verbal learning [35]. Furthermore, Foster et al. [36] reported the former to be correlated with executive function and verbal memory in healthy subjects aged between 53 and 89 years. An attenuated MMN amplitude to duration deviants may therefore not be specific to schizophrenia but to deficits of cognitive function.

In summary, MMN amplitude decrement seems to be associated with poor neuropsychological functioning, particularly of executive function and tasks targeting verbal domains. However, correlations between MMN amplitude and cognitive deficits have not been reported consistently. It is, in any case, notable that previous studies reporting a relationship between MMN amplitude and neuropsychological function have been carried out with duration deviant stimuli leaving open the question whether similar results can be expected from frequency deviant stimuli.

MMN latency is determined by the time at which the deviant stimulus can be distinguished from the standard stimulus [37]. In contrast to the highly reliable finding of diminished MMN amplitude, the analysis of MMN latency has not been established to play a crucial role in schizophrenia-related abnormality of early sensory information processing. Shortened MMN latency has been found to be related to an increased magnitude of stimulus change in healthy controls [38], [39] as well as in schizophrenia patients [40]. Furthermore, there is evidence that complex stimuli, high stimulus presentation rate and the number of standards preceding a deviant stimulus shorten MMN latency [41]–[46]. Recent publications provided empirical evidence for MMN latency to be sensitive to nicotine [47]–[49] and alcohol [50], [51] administration. The former has also been found to moderate MMN latency to duration deviants in schizophrenia patients [52]. There are some additional characteristics that have been associated with shortened MMN latencies such as high IQ [53], high musical experience [54] and good performance in 2nd language learning [55]. However, the relationship between intellectual disability and MMN latency was not always confirmed [56], [57]. High age may be associated with prolonged latency, as well as Alzheimer dementia, Asperger syndrome and specific language impairment [58]–[60] whereas younger subjects may show reduced MMN latency [61].

Surveys of the MMN literature do not report consistent latency differences between patients and controls. Some authors reported a prolonged latency [51], [62], [63], while others did not [23], [64], [65]. However, recent studies provided evidence of shortened MMN latency to frequency [40] and duration deviants [66] in schizophrenia patients relative to healthy controls. The latter have also been reported in a magneto-encephalographic (MEG) experiment in subjects with ultra high risk for schizophrenia (UHR) [67].

Currently, very little is known about the relationship between the clinical/cognitive status and MMN latency. Grzella et al. [18] reported severity of positive symptoms to be associated with shortened latency in schizophrenia patients receiving antipsychotic medication. However, a recent study [68] found MMN latency to be positively correlated to the negative and global psychopathological subscales of the Positive and Negative Syndrome Scale (PANSS) [69]. Toyomaki et al. [27] reported that schizophrenia patients with poor working memory revealed a prolonged MMN latency to duration deviants. Owing to the discrepant results, no conclusions can so far be drawn regarding the relationship between clinical symptoms or neuropsychological function and MMN latency to duration and frequency deviants.

The present study aimed to investigate whether MMN amplitude and latency to frequency and duration deviant tones are associated with clinical symptoms and measures of cognitive function in schizophrenia patients. We expected to replicate recent findings of the relationships between MMN and clinical symptoms of schizophrenia in particular, positive symptoms.

## Methods

### Participants

A total of 56 participants took part in the study, among them 40 patients diagnosed with schizophrenia according to DSM-IV-TR [70] and 16 healthy control subjects. Additional 3 patients had to be excluded due to technical problems. Patients and controls were matched for age, verbal intelligence, gender proportion and years of education. Subject characteristics are shown in Table 1.

**Table 1:** Demographic, clinical and neuropsychological characteristics of schizophrenia patients and healthy controls.

	Patients n = 40 (mean, (SD))	Controls n = 16 (mean, (SD))	Statistic (p-value)
Gender, N (f, m)	14 f, 35%; 26 m, 65%	6 f, 37.5%; 10 m, 62.5%	χ^2^_1, 56_ .03 (.860)
Age, y	38.72 (10.59)	38 (10.65)	*t*_1, 54_ = .23 (.818)
Education, (y)	13.24 (4.02)	13.72 (2.93)	*t*_1, 54_ = −.50 (.623)
MWT-B (score)	25.50 (4.72)	26 (4.52)	*t*_1, 54_ = −.36 (.718)
PANSS positive (score)	11.80 (3.72)	/	/
PANSS negative (score)	13.30 (6.24)	/	/
PANSS global (score)	27.95 (10.99)	/	/
PANSS overall (score)	53.05 (19.23)	/	/
SOFAS Scale (score)	46.40 (9.15)	/	/
GAF Scale (score)	45.50 (8.84)	/	/
Duration of Illness (y)	14.31 (9.25)	/	/
Trail Making Test-A (sec.)	43.48 (22.19)	26.62 (6.16)	*t*_1,50.6_ = 4.40 (<.001)
Trail Making Test-B (sec.)	112 (45.61)	70.94 (29.59)	*t*_1,54_ = 3.33 (.002)
Verbal Fluency (N correct)	28.58 (8.43)	37.69 (10.92)	*t*_1,54_ = −3.35 (.001)
Digit Symbol Test (N correct)	38.87 (11.61)	57 (10.39)	*t*_1,54_ = −5.42 (<.001)
Logical Memory Test, immediate (score)	14.55 (6.94)	20.62 (9.05)	*t*_1,54_ = −2.71 (.009)
Logical Memory Test, delayed (score)	9.62 (6.73)	15.25 (7.99)	*t*_1,54_ = −2.68 (.010)
Digit Span Test, forward (score)	6.78 (1.83)	8.44 (2.03)	*t*_1,54_ = −2.97 (.004)
Digit Span Test, backward (score)	5.02 (1.64)	7 (2.16)	*t*_1,54_ = 3.71 (<.001)

Inclusion criteria of patients were the absence of other major psychiatric or neurological disorders as well as of hearing impairment, an age between 18 and 54 years and an IQ>70. Only patients with stable doses of antipsychotic medication were included and participants using benzodiazepines were excluded. According to DSM-IV-TR [70], twenty-one of the patients had a diagnosis of schizophrenia of the paranoid type, 5 of the disorganised, 5 the residual and 2 of the undifferentiated type. Seven patients had a diagnosis of schizoaffective disorder. Twenty-seven patients received solely atypical antipsychotic medication, 4 patients typical and 9 received both. Nine of the patients also received antidepressant medication. Onset of the illness was at a mean age of 24.28 years (SD = 8.33) and ranged between 14 and 47 years. The study was approved by the ethics committee of the University of Duisburg-Essen. All participants and their legal representative (if applicable) gave their written informed consent before being included. Potential participants who declined were not disadvantaged with regard to clinical treatment. Participants received a remuneration.

### MMN Stimuli

Stimuli consisted of 2400 binaural tone pips administered via headphones with a SOA of 500 ms. Standard sine-wave tones (78.04% probability, i.e., 1872 trials) were 1 kHz, 80 dBA and 80 ms in duration with rise and fall times of 10 ms. Frequency deviant tones were 1.2 kHz (10.98% probability, i.e., 263 trials) and duration deviant tones were 40 ms in duration (10.98% probability, 263 trials; rise and fall times 5 ms) with all other stimulus parameters being identical, respectively. Stimuli were presented in random order while participants watched a silent nature film (visual angle 5°) using the Software Presentation V14.1 (Neurobehavioral Systems Inc.).

### EEG recording

Participants were seated in a comfortable chair and were instructed to avoid movement and keep their eyes fixated on the video screen. They were given a short rest after presentation of half of the stimuli.

Electroencephalographic activity was recorded from the scalp via 28 Ag/AgCl electrodes in preconfigured caps (Easycap). Electrode sites included those of the extended International 10–20 System. Linked earlobes were used as the reference and the forehead at AFz was used as ground. EOG was recorded medially above and below the right eye and at the outer canthi of the eyes to monitor vertical and horizontal eye movements. Electrode impedances were kept below 5 kΩ at all sites. DC-coupled amplifiers (Brain Amp DC, Brain Products Ltd., Munich) were used with a band pass filter of DC = 0 Hz to 250 Hz and a digitisation rate of 500 Hz. Averaging and artefact rejection were performed offline.

### Data preprocessing

Trials with artefacts due to muscular activity and complex eye movement (when subjects looked around) were excluded during a first visual inspection of the raw data by an experienced scientific research associate. Data were then passed through an IIR Butterworth filter extending from 0.1 to 30 Hz and a 50 Hz notch filter before artefact correction [71]. Artefacts due to ocular movement were corrected by independent component analysis [72]. Segments of MMN-related indices were determined within an epoch extending from 100 ms before to 400 ms after stimulus onset. Epochs were baseline-corrected by subtracting the average prestimulus voltage 100 ms before stimulus onset. Epochs exceeding +/−50 µV were rejected. Single subject averages were computed for segments with standard and deviant stimuli. MMN difference waves were obtained by subtracting single subject averages to standard from those to deviant tones. MMN amplitude and peak latency was measured between 100 and 300 ms at electrode sites Fz, FCz and Cz and further processed using statistical software (SPSS v 20, IBM Inc.). Finally, grand average curves were computed for duration and frequency MMNs in patients and controls separately with regard to each electrode site.

### Clinical interviews and neuropsychological measures

The diagnosis of schizophrenia was confirmed by means of the *Structured Clinical Interview (SCID) for DMS-IV* [73] and the *Positive and Negative Syndrome Scale* [69]. Additionally, the *Global Assessment of Functioning Scale* [74] and the *Social and Occupational Functioning Assessment Scale* [75] were used. In both scales, lower scores indicate more severe symptoms and greater disability. Control participants were administered the *Diagnostic Interview for Psychological Disorders* [76] to ascertain the absence of major psychiatric disorders.

Neuropsychological tests included the *Multiple Word Recognition Test* (MWT-B; Lehrl, 1989) which is considered a measure of premorbid verbal intelligence. The *Word Fluency Test* [77] was used to asses verbal fluency. Visual processing speed and motor implementation of visual information was assessed using the *Trail Making Test* [78] part *A* (TMT A) with cognitive switching or flexibility assessed by part B (TMT B). Furthermore, two subtests from the German version of the Wechsler Adult Intelligence Scale [79] were employed, the Digit Symbol Test to assess figural processing speed and the Digit Span Test for verbal working memory. Finally, the *Wechsler Logical Memory (Prose Recall) Test* [80] was used (immediate and delayed retrieval) to assess auditory verbal memory [81].

### Statistical analyses

Neuropsychological test scores were compared between groups by means of two sample *t*-tests or Welch tests in case of heterogeneous between group variances, respectively. To account for dependencies between electrode sites, MMN amplitude and peak latency were submitted to repeated measures ANOVA with a 2×2×3 (group×deviant×electrode site [Fz/Fcz/Cz]) design. In cases of the violation of the assumption of sphericity, Greenhouse Geisser corrected p-values for the F-statistics are reported. Unequal sample sizes and heterogeneous variances between groups may lead to progressive decisions in favour of the H1 [82]. Therefore, we subsequently inspected significant repeated measures ANOVA effects by means of the Welch test (Table 1), when heterogeneous variances are assumed. Moreover, in order to prevent the occurrence of type-I errors, our results derived from the repeated measures ANOVA were solely considered to be significant, when surviving an alpha threshold of p≤.01.

Spearman rank correlations between MMN parameters (amplitude and peak latency) and clinical ratings as well as neuropsychological variables were carried out in patients only. As independence between electrode sites is not given in ERP analysis, applying the Bonferroni method would yield an overly conservative rejection of relevant associations. To take into account the alpha error accumulation and to incorporate dependencies of adjacent electrode sites, correlations between MMN amplitude and latency and clinical or neuropsychological variables were interpreted as statistically significant only above the threshold of p≤.05 for at least two of three adjacent midline electrodes (Fz, FCz and Cz). To assess the effect of nicotine status on MMN parameters, repeated measures ANOVAs were conducted within the patient group.

## Results

### Neuropsychological tests (Table 1)

Group comparisons were carried out with one-way ANOVAs. Compared to healthy controls, schizophrenia patients showed an impaired performance with regard to all neuropsychological tests apart from verbal IQ.

### Mismatch negativity

The following mean number and standard deviation of segments were included in the determination of MMN: standard tone – controls: 1864.8 (SD = 102.4), patients: 1875.2 (68.3); frequency deviant - controls: 204.8 (11.3), patients: 205.6 (8.1); duration deviant – controls: 204.5 (12.0), patients: 206.2 (6.9). There were no significant group differences. MMN amplitude and latency were submitted separately to repeated measures ANOVA with a 2×2×3 (Group [patients/controls]×Type of deviant [duration/frequency]×Electrode [Fz/FCz/Cz]) design. Group means and SD of MMN peak amplitude and latency are shown in Table 2.

**Table 2:** Mean (SD) amplitudes and latencies to duration and frequency deviants for schizophrenia patients and healthy controls.

Deviant	Location	Group				Statistic (p-value)	
		Patients (N = 40)		Controls (N = 16)			
		µV	ms	µV	ms	µV	ms
frequency	Fz	−2.79 (1.62)	177.20 (29.07)	−4.32 (2.23)	193.13 (22.73)	*t*_1, 54_ = 2.86 (.006)	*t*_1, 35.24_ = −2.18 (.036)*
	FCz	−2.74 (1.60)	179.75 (30.83)	−4.15 (2.22)	194.00 (22.46)	*t*_1, 54_ = 2.66 (.010)	*t*_1, 37.85_ = −1.92 (.063)*
	Cz	−2.27 (1.45)	177.65 (29.44)	−3.63 (2.05)	195.75 (24.15)	*t*_1, 54_ = 2.80 (.007)	*t*_1, 54_ = −2.18 (.034)
duration	Fz	−3.24 (1.33)	208.80 (30.74)	−5.11 (2.66)	230.63 (16.70)	*t*_1, 18.06_ = 2.68 (.015)*	*t*_1, 48.77_ = −3.41 (<.001)*
	FCz	−3.25 (1.52)	206.25 (34.22)	−5.3 (2.79)	232.00 (15.78)	*t*_1, 18.66_ = 2.77 (.012)*	*t*_1, 52.73_ = −3.84 (<.001)*
	Cz	−2.96 (1.42)	205.65 (33.32)	−4.67 (2.44)	230.86 (13.56)	*t*_1, 19.17_ = 2.62 (.017)*	*t*_1, 53.94_ = −4.03 (<.001)*

Between-group t-statistics (p-values) are reported for amplitude (µV) and latency (ms) separately for each electrode. Adjusted t-statistics (Welch-test) are reported (*), in the case of unequal variances.

#### Amplitude (Figure 1 and 2)

Repeated-measures ANOVA revealed a main effect for group with regard to MMN peak amplitude (F_1, 54_ = 12.17, p = .001). Compared to healthy controls, patients showed diminished MMN amplitude with regard to both deviants. There was also a significant main effect for electrode showing a decrease of negativity from Fz to Cz (F_2, 80.43_ = 39.97, p<.001). MMN amplitude to duration deviants was found to be increased compared to frequency deviants, resulting in a significant main effect for type of deviant (F_1, 54_ = 11.93, p = .001). Moreover, as shown in Figure 3, group differences were more pronounced for duration than frequency deviants. Within the patient group, subsequent repeated measures ANOVA identified significantly increased MMN amplitude in smokers compared to non-smokers with regard to both deviants (F_1, 38_ = 10.79, p = .002). Furthermore, there were significant main effects for type of deviant (F_1, 38_ = 6.11, p = .018) and electrode (F_1.5, 38_ = 19.48, p<.001).

**Figure 1:**
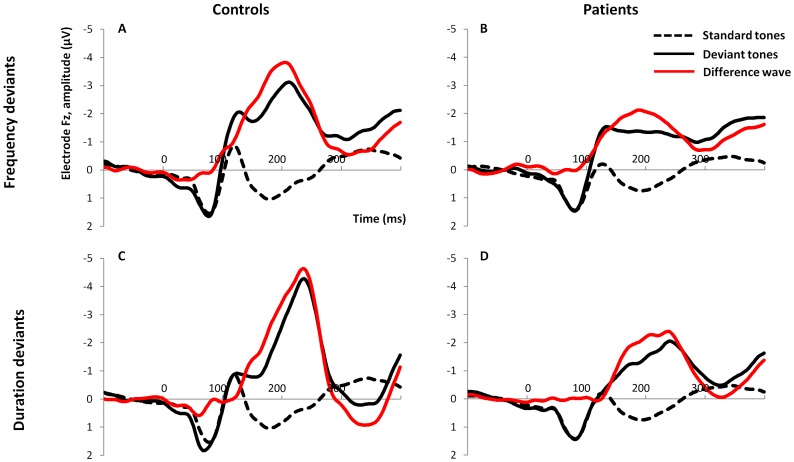
Grand averages of responses to standard and deviant tones and the resulting difference waveforms. Top: Frequency deviants in controls (A) and patients (B); bottom: Duration deviants in controls (C) and patients (D).

**Figure 2:**
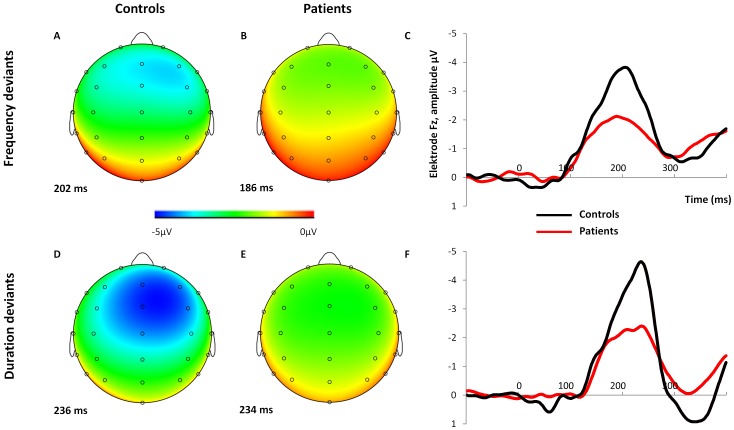
28 channel EEG amplitude distributions and comparison of the between-group MMN waveforms. Scalp electrode maps of MMN to frequency deviant (A, B) and duration deviant tones (D, E) in controls (A, D) and patients (B, E). MMN waveforms of patients and controls to frequency (C) and duration deviant tones (F), both at Fz are shown on the right.

**Figure 3:**
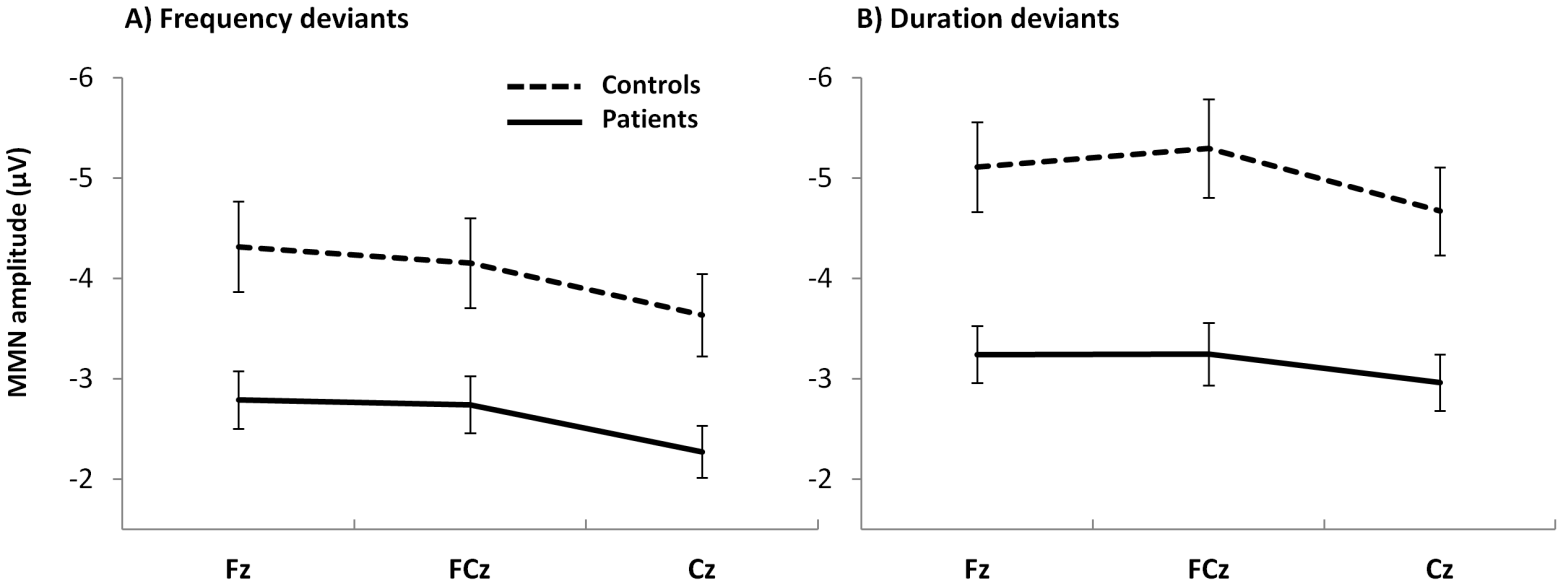
Diagrams of group means and standard errors. MMN amplitudes to frequency (A) and duration deviants (B) are presented for both groups at midline electrode sites.

#### Latency (Figure 1 and 2)

Between group analysis revealed significantly shortened MMN latency to both deviants in schizophrenia patients as compared to healthy controls (F_1, 54_ = 9.41, p = .003). There was also a significant main effect for type of deviant (F_1, 54_ = 42.58, p<.001) indicating prolonged latency to duration as compared to frequency deviants. Nicotine dependency did not affect MMN latency.

### Relations between variables

#### Neuropsychology

**Figure 4:**
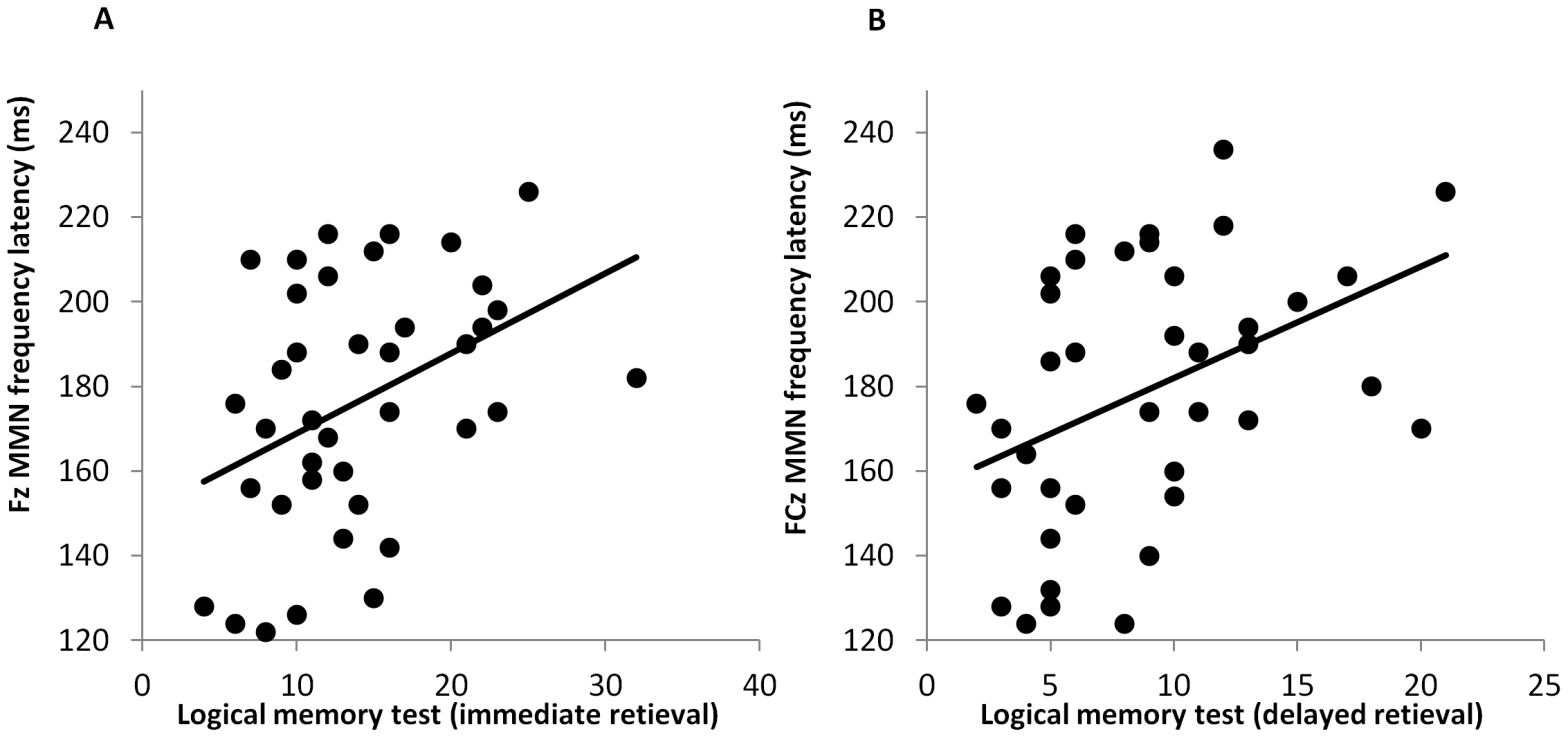
Linear associations between MMN latency and patient's verbal memory performance. Scatterplots of immediate (A) and delayed (B) verbal memory performance score against MMN amplitude to frequency deviants at Fz and FCz, respectively.

Correlations were computed between MMN parameters and neuropsychological test performance in the patient group after removal of one outlier. MMN latency to frequency deviants was significantly correlated with immediate (Fz Spearman's r = .403, p = .011, Figure 4A; FCz r = .379, p = .017; Cz r = .348, p = .030) and delayed retrieval (Fz r = .384, p = .016, Figure 4B; FCz r = .440, p = .005; Cz r = .380, p = .017) of the logical memory test. The longer the frequency deviant latency the more prose elements were remembered by patients. Details of the correlations between MMN parameters and neuropsychological test variables can be found in Table S1.

#### Clinical variables

**Figure 5:**
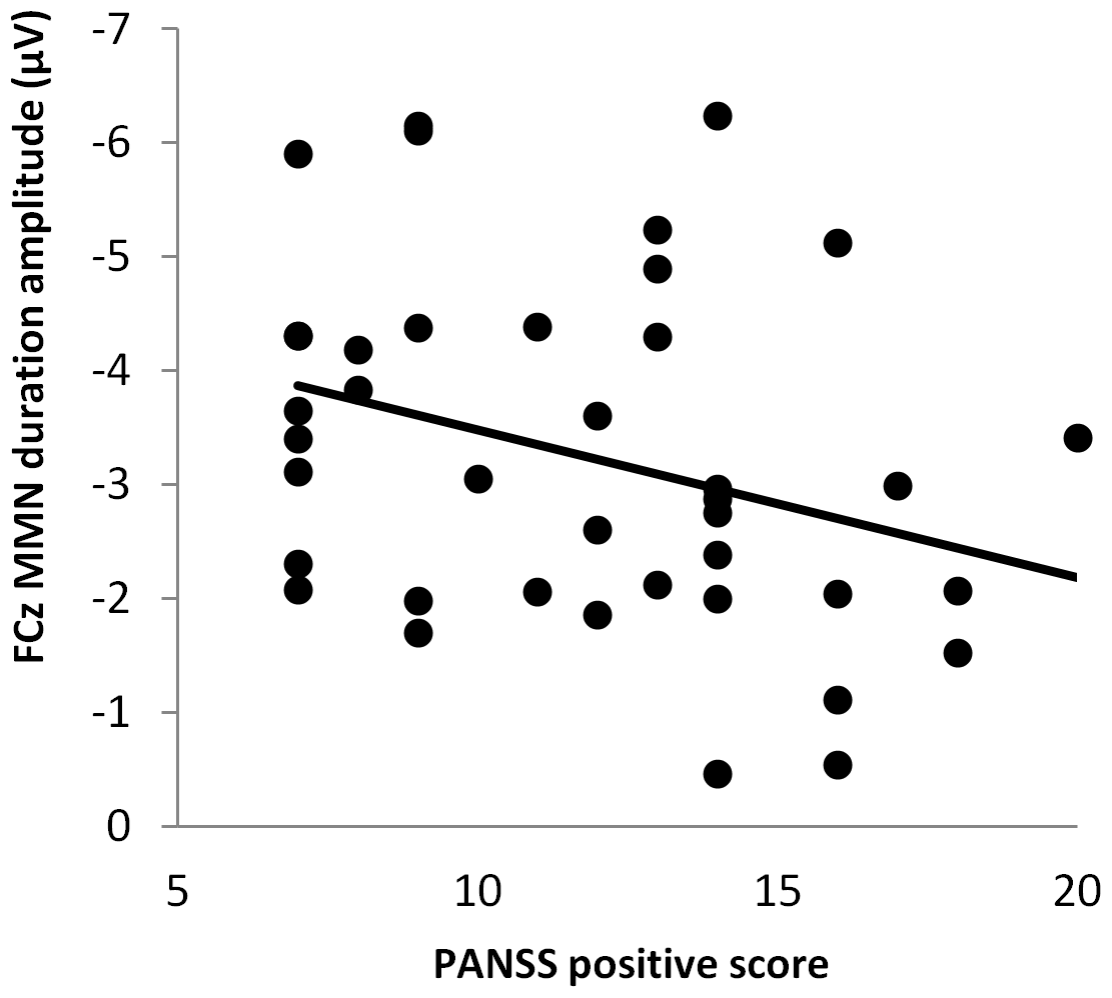
Positive symptom scores and MMN amplitude. Scatterplot of PANSS positive symptoms against MMN amplitude to duration deviants at FCz.

Spearman rank correlations were computed between MMN parameters and PANSS symptom ratings. MMN amplitude to duration deviants was significantly correlated with PANSS positive score at two of three midline electrode sites (Fz Spearman's r = .285, p = .074; FCz r = .338, p = .033, Fig. 5; Cz r = .342, p = .031), i.e. patients with more pronounced positive symptoms showed a decreased (less negative) MMN amplitude (Figure 5).

#### Amplitude and latency

Spearman rank correlations were computed between MMN amplitude and latency. There were no significant results.

#### Duration of illness

MMN amplitude to frequency deviants was significantly correlated with duration of illness (Fz Spearman's r = .305, p = .059; FCz r = .322, p = .008; Cz r = .326, p = .043), i.e., higher MMN amplitudes to frequency deviants were associated with shorter duration of illness. No correlations between MMN parameters and age were found.

## Discussion

While MMN amplitude decrement may be considered a highly reliable finding in schizophrenia, results with regard to MMN latency have been reported less often. In the present study MMN amplitude to both frequency and duration deviants were diminished in patients compared to controls. Furthermore, compared to controls, patients showed a shorter MMN latency to duration and frequency deviants. Only the latter was related to poor verbal memory performance. Increased duration of illness was associated with decreased MMN amplitude to frequency deviants. Additionally, decreased MMN amplitude to duration deviants was correlated with a higher PANNS positive score in schizophrenia patients.

MMN latency to both types of deviants was shortened in schizophrenia patients as compared to controls in the present study, whereas others failed to find significant group differences [7], [68], or else they reported a prolonged MMN latency in patients [51], [83]. Recently, Horton et al. [40] reported MMN latency to frequency deviants to be shorter in schizophrenia subjects and showed that it was progressively shortened with increased magnitude of stimulus change as has previously been shown to be the case for healthy controls with respect to frequency [39] and duration deviants [38]. Domján et al. [66] observed shorter latency to duration deviants in schizophrenia patients. Corroborative evidence has been reported in a MEG study with subjects at ultra-high-risk for schizophrenia [67]. In summary, our results are in line with reports by Horton [40] and Domjan [66], but in contrast to those of many others.

Cognitive deficits have been found to be a core feature in schizophrenia [24], [84]. MMN amplitude has previously been reported to be related to executive function [27] and verbal memory [30] in schizophrenia, which our study failed to confirm. Instead, verbal memory retrieval was related to MMN latency to frequency deviants. Correlations between neuropsychological measures and MMN latency have hardly ever been reported in schizophrenia. One study by Toyomaki and colleagues [27] found prolonged MMN latency to duration deviants to be associated with executive function deficits. To our knowledge, the present study is the first one reporting a relationship between prolonged MMN latency and enhanced memory function.

Based on their findings of a shortened MMN latency being correlated with more pronounced positive symptoms, Grzella et al. [18] suggested that a shortened MMN latency in schizophrenia may indicate dysfunctional rapid early stimulus processing which may induce errors in the course of integrating the obtained information into existing neural networks. A similar argument of dysfunctional bottom-up processing of sensory input was put forward by Javitt [85] who emphasized its important (but not exclusive) role in contributing to cognitive impairments in schizophrenia. He proposed a bottle-neck model: Inaccurate early sensory stimulus processing may lead to restricted cognitive performance at a later stage. In an attempt to accommodate findings of MMN deficits, Todd et al. (2012) proposed that it is not the ‘prediction error generation detection, but rather the estimation of error size that is dysfunctional in schizophrenia [86], [87]. This was supported by studies assessing for the discrimination ability of auditory sensory input, resulting in correlated deficits of behavioral performance and MMN measures [88], [89]. It is suggested that the difference between standard and deviant tone events are not accurately registered and their representations therefore largely overlapping [87].

Both groups showed a longer MMN latency to duration compared to frequency deviant tones. MMN latency has been suggested to indicate the time of identifying the difference between standard and deviant tone representations [37]. From a theoretical point of view, shortened MMN latency may indicate a briefer engagement of a comparator process [40]. However, from a more biological perspective and with regard to the adaptation hypothesis of the MMN [8], latency may be interpreted in terms of temporal responsiveness of the corresponding neuronal populations of the auditory cortex. Cortical frequency representations are thought to be processed at multiple and therefore partially redundant tonotopically organized maps in primary and secondary auditory cortex. In contrast, duration representations are more widely distributed and computationally complex as has been suggested by He [90], [91]. Furthermore, studies evaluating MMNm sources [92], dipole modeling [93], PET [94] and fMRI [95] provided converging evidence of differential sensitivity of cortical fields in superior temporal regions with respect to different types of auditory change in healthy controls.

Patients who smoked were found to have significantly enhanced MMN peak amplitudes as compared to non smoking patients in the present study. This finding is in line with those of other studies [48], [52] reporting an ameliorating effect of nicotine to MMN amplitude in schizophrenia patients. Nicotine has a cholinergic effect which has been reported to affect a wide variety of cognitive functions such as perception, selective attention, associative learning and memory [96]–[98]. In schizophrenia, the rate of smoking patients is up to four-fold higher relative to the rate seen in the general population which has been interpreted to serve as a form of self-medication [99]. However, previous studies assessed the effect of acute nicotine administration whereas patients of the present study had not smoked for some 30 min which may have constituted a withdrawal. While our study was not primarily designed to assess nicotine effects, the data underline the necessity of taking smoking behavior in the assessment of MMN into account. Previous studies also demonstrated MMN latency to be sensitive to nicotine status in healthy controls [47], [49] as well as in schizophrenia patients [48], which was, conversely, not corroborated by our findings.

Patients with more pronounced positive symptoms showed a decreased MMN amplitude to duration deviants in the present study which has been reported previously [13], [21], [23]. But there are also reports of a correlation between MMN amplitude and negative symptoms [17], [88]. There were no significant correlations with MMN latency and clinical symptoms in the present study unlike in that of Grzella et al. [18] who found MMN latency to complex novel deviants negatively correlated with positive symptoms, i.e., a shorter latency was associated with more pronounced symptoms. The discrepant results may be due to the heterogeneity between patient samples, the used deviant types, the kind of oddball paradigm (‘classic’ or ‘optimal feature paradigm’) the source of patients (whether inpatients or outpatients), and the range of symptom ratings within the sample.

A limitation of the present study is related to the unequal sample size between groups which affects the statistical power of effects. Therefore, we focused on the within group correlations in the patient group and did not report associations in the control group. Another limitation is inherent to the limited knowledge on subcomponents contributing to the generation of the resulting MMN. Alterations in the expression of different sub-components of the MMN may result in latency shifts. And while the recent review by Bendixen et al. [10] outlines processes that may form independent contributions to MMN generation, more has to be known as to reliably identify and separate these in the data.

In conclusion, our study replicates the finding of decreased MMN amplitude with respect to both frequency and duration deviants in schizophrenia. Relating them to clinical measures, lower MMN amplitude to duration deviants was significantly associated with more severe PANSS positive symptoms. Furthermore, our finding of a shorter MMN latency in patients as compared to controls and its relationship with verbal memory performance, emphasize the importance of temporal information in early auditory discrimination processing in schizophrenia.

## Supporting Information

Table S1 Spearman rank correlations (p-values) between neuropsychological measures and MMN parameters in schizophrenia patients (n = 40). Abbreviations: LM = logical memory test (immediate retrieval), LM (delay) = logical memory test (immediate retrieval), TMT (A and B) = Trial Making Test (Version A and B), FAS = verbal fluency test, DST = digit symbol test, DS (f) = digit span test (forward), DS (b) = digit span test backwards. ^*^ = p≤.05. ^**^ = p≤.01.
(DOCX)
