# Supplementary material for: Mismatch Negativity Latency and Cognitive Function in Schizophrenia
Source: PLoS One. 2014 Apr 16;9(4):e84536. doi: 10.1371/journal.pone.0084536 (PMC3989165; doi:10.1371/journal.pone.0084536)
Supplement: Table S1 — Spearman rank correlations (p-values) between neuropsychological measures and MMN parameters in schizophrenia patients (n = 40). Abbreviations: LM = logical memory test (immediate retrieval), LM (delay) = logical memory test (immediate retrieval), TMT (A and B) = Trial Making Test (Version A and B), FAS = verbal fluency test, DST = digit symbol test, DS (f) = digit span test (forward), DS (b) = digit span test backwards. * = p≤.05. ** = p≤.01. (DOCX) [file pone.0084536.s001.docx]

| **Deviant** | **MMN** | **Electrode** | **Neuropsychological Measure, Spearman's r (p-Value)** | | | | | | | |
| --- | --- | --- | --- | --- | --- | --- | --- | --- | --- | --- |
|  |  |  | LM | LM (delay) | TMT A | TMT B | FAS | DST | DS (f) | DS (b) |
| Duration | Latency | Fz | .013 (.936) | .077 (.643) | .195 (.235) | .079 (.633) | -.138 (.401) | -.169 (.312) | -.229 (.161) | -.234 (.151) |
|  |  | FCZ | -.013 (.937) | -.016 (.922) | .198 (.227) | .013 (.935) | -.125 (.450) | -.180 (.281) | -.201 (.220) | -.257 (.115) |
|  |  | Cz | -.028 (.866) | -.063 (.705) | .233 (.153) | .032 (.847) | -.166 (.313) | -.177 (.289) | -.242 (.138) | -.287 (.077) |
|  | Amplitude | Fz | .001 (.993) | -.063 (.704) | -.080 (.627) | -.120 (.467) | .242 (.138) | .150 (.369) | .177 (.280) | .268 (.099) |
|  |  | FCZ | -.026 (.877) | -.074 (.654) | -.006 (.969) | -.040 (.810) | .178 (.277) | .099 (.556) | .100 (.544) | .180 (.274) |
|  |  | Cz | -.070 (.672) | -.092 (.577) | -.007 (.965) | -.025 (.879) | .140 (.397) | .108 (.518) | .016 (.922) | .168 (.307) |
|  |  |  |  |  |  |  |  |  |  |  |
| Frequency | Latency | Fz | .403 (.011)* | .384 (.016)* | .120 (.467) | -.006 (.973) | .023 (.888) | -.203 (.222) | .001 (.994) | .002 (.991) |
|  |  | FCZ | .379 (.017)* | .440 (.005)** | .064 (.698) | .024 (.883) | -.038 (.820) | -.188 (.259) | -.057 (.729) | -.032 (.847) |
|  |  | Cz | .348 (.030)* | .380 (.017)* | .157 (.341) | .047 (.775) | -.047 (.775) | -.230 (.166) | .009 (.957) | -.031 (.849) |
|  | Amplitude | Fz | -.254 (.119) | -.222 (.174) | .-144 (.383) | .042 (.798) | .131 (.427) | .063 (.708) | .003 (.986) | .177 (.281) |
|  |  | FCZ | -.262 (.107) | -.227 (.164) | -.154 (.349) | .081 (.624) | .090 (.584) | .092 (.584) | .026 (.876) | .172 (.296) |
|  |  | Cz | -.245* (.132) | -.209 (.202) | -.117 (.477) | .095 (.565) | .113 (.493) | .085 (.611) | .021 (.899) | .161 (.326) |
